# Synthesis of Green and Red-Emitting Polymethyl Methacrylate Composites Grafted from ZnAl_2_O_4_:Mn-Bonded GO via Surface-Initiated Atom Transfer Radical Polymerization

**DOI:** 10.3390/polym14173689

**Published:** 2022-09-05

**Authors:** Ming Gao, Chi-Fai Cheung, Bo Wang, Chunjin Wang

**Affiliations:** State Key Laboratory of Ultra-Precision Machining Technology, Department of Industrial and Systems Engineering, The Hong Kong Polytechnic University, Hung Hom, Kowloon, Hong Kong, China

**Keywords:** dual photoluminescent, polymer composites, polymethyl methacrylate, green and red-emitting, surface-initiated atom transfer radical polymerization

## Abstract

A novel dual green and red-emitting photoluminescent polymer composite ZnAl_2_O_4_:Mn-bonded GO/polymethyl methacrylate (PMMA) was synthesized in a single-step reaction by surface-initiated atom transfer radical polymerization (SI-ATRP). The polymer chain was surface-initiated from the ZnAl_2_O_4_:Mn/GO, and the final products have a homogenous photoluminescent property from ZnAl_2_O_4_:Mn and better mechanical properties strengthened by graphene oxide (GO). The morphologies of ZnAl_2_O_4_:Mn/GO and the polymer composites were verified by scanning electron microscopy (SEM) and transmission electron microscopy (TEM). X-ray diffraction analysis (XRD) revealed the two valence states of Mn (Mn^2+^, Mn^4+^) existing in the ZnAl_2_O_4_ host lattice, while Fourier-transform infrared spectroscopy (FTIR) spectra proved the transference of the active group, C-Br, from the initiator to the monomer during the polymerization. Gel permeation chromatography (GPC) shows the narrow dispersity of polymer composites fabricated through SI-ATRP. The SEM and FTIR results show the successful ‘graft’ of the polymer chains from the surface of ZnAl_2_O_4_:Mn/GO. The dual green and red-emitting polymer composites were synthesized, confirmed by the photoluminescence (PL) and photoluminescence excitation (PLE) results.

## 1. Introduction

In light of the current growth of the world’s population, by 2050, the global population will have risen to 9 billion, 70% of which will be living in urban areas [1,2]. To solve the food shortage problem, plant factories have attracted increasing attention. A plant factory is “an indoor vertical farming system for efficient quality food production” whose particular emphasis is on plant factory artificial lights (PFAL) [2]. The indoor system has mainly two types of light sources: solely artificial light and artificial light plus sunlight. Most artificial lights used in plant factories are commercial light emitting diode (LED) lights which are produced by coating yellow phosphors (YAG: Ce^3+^, (Y, Gd)_3_(Al, Ga)_5_O_12_:Ce^3+^) on blue LED chips, causing problems of a highly correlated colour temperature and low colour rendering index [3,4,5]. This kind of white light emitting diode (WLED) lacks red light, which is crucial for the growth of plants. To solve this limitation, phosphors containing red light-emitting elements are essential. Most recent red-emitting phosphors are fabricated by adding rare-earth ions like Eu^2+^ to nitride due to their stable photoluminescent properties [6,7,8]. However, the fabrication of Eu-doped materials needs a high synthesis temperature (≥1800 °C), high pressure, and usually a nitrogen atmosphere [9]. To overcome these problems, non-rare-earth phosphors doped by transition metal elements (Mn, Co) have attracted rising attention. The selected phosphor in this project was a non-rare-earth manganese-doped zinc aluminate oxide (ZnAl_2_O_4_:Mn). Manganese is a promising replacement material that has been paid increasing attention to nowadays; it can emit green and red light at wavelengths from 490 nm to 750 nm with high optical performance. Compared with rare-earth elements, manganese is environmentally friendly, has easier synthesis conditions, and is costly [10,11]. In the AB_2_O_4_ structure, A usually refers to a divalent metal cation in a tetrahedral position surrounded by four oxygen ions, while B refers to a trivalent cation in the octahedral void caused by six oxygen ions [12,13]. Among these spinel-structured materials, zinc aluminate is widely used in many catalytic reactions because of its excellent thermal stability, hydrophobicity, and low surface acidity [11]. Moreover, ZnAl_2_O_4_ is considered an excellent photoelectric material due to its wide bandgap of 3.8 eV. It is one of the spinel-type oxide materials with the AB_2_O_4_ formula, which has good thermal and chemical stability, high mechanical resistance, and satisfactory fluorescence efficiency [14]. The structure and photoluminescence of ZnAl_2_O_4_ can be adjusted by doping different elements into the crystal structures. For these reasons, Mn-doped ZnAl_2_O_4_ was selected in this research. 

Polymers are materials that have been widely used in advanced areas for many years due to their versatility. However, a single polymer usually cannot achieve all requirements. As a result, polymer composites have been selected [15]. Recently, most polymer composites with photoluminescent properties have been fabricated through physical methods such as coating, solution casting or solution blending [16,17,18]. Sumara Khursheed has made green emitting SrAl_2_O_4_:Eu^2+^, Dy^3+^ embedded PMMA nanocomposites through a solution casting method [19]. Jai Prakash also reported a green emitting nanocomposite, ZnO:Tb^3+^/PS, using a solution casting method [20]. Long-Xiang Cheng fabricated an inorganic phosphor/polymer composite film with a self-repairing property [21]. Mokni Marwa produced a new light transducer poly (vinylidene fluoride-trifluoroethylene-chlorotrifluoroethylene) (P(VDF-TrFE-CTFE))/phosphor composite by casting a polymer composite on an ITO electrode [22]. However, these methods have problems with mixing inhomogeneous and solvent-depending fabrication conditions. To overcome these limitations, synthesizing a kind of controllable photoluminescent polymer composite is a promising strategy, and atom transfer radical polymerization (ATRP) is an excellent choice. ATRP is one of the widely used effective polymerization methods of controlled radical polymerization (CRP). This method allows researchers to put different monomers and specific functionalities with defined properties together in a controllable process [23]. In ATRP, the active species is formed by a reversible redox process with a transition metal complex catalyst. The process is one-electron oxidation, which also attracts a halogen atom from a dormant material. With the development of ATRP, Yao et al. designed an in situ process to fabricate a PMMA-based composite which was initiated by modified carbon nanotubes [24]. Baskaran reported the polymerization of polystyrene (PS) and PMMA from multi-wall carbon nanotubes by the ATRP method with tunable polymer molecular weight, which was achieved by adjusting the proportion between initiator-modified nanotubes and the monomers [25]. Liu et al. reported a copolymer of linear PS and poly(N-isopropyl acrylamide) using the same strategy [26]. Therefore, in these strategies, carbon nanotubes were selected as the connection between polymer chains, where the existence of carbon materials improves the mechanical properties and thermal stability of polymers. 

According to the polymerization process mentioned above, graphene oxide was selected as the modified “grafting” initiating species, allowing polymer chains to grow from its surface. The oxidation groups on the graphene oxide surface make the modification process of the initiator easy. Graphene is a 2D material with superior large relative surface properties. Although the bandgap of graphene is zero, which makes it a great challenge to employ in luminescent applications, a chemical-derived GO is a 2D network with both sp2 and sp3 atoms which have sufficient conductivity caused by the electron-hole pair recombination localized in sp2 carbon clusters within the sp3 matrix [27,28]. Additionally, GO/PMMA composites were used as reinforcement in PMMA, as reported by Gil Concalves. In this research work, GPMMA was fabricated through ATRP in which PMMA chains were ‘grafted’ onto the surface of GO. The functional groups on the surface of GO improve the interfacial adhesion between filters and the composites, which has the benefit of transferring stress from the polymer to the filters. The GPMMA filters can improve the mechanical properties of pure PMMA significantly [29]. 

This project describes a novel kind of photoluminescent polymer composite by surface-initial atom transfer radical polymerization (SI-ATRP) on the graphene oxide surface, which contains green and red dual light-emitted phosphors. The fabricated polymer composites have narrow dispersity and photoluminescent properties, giving them a wide application in plant factories and greenhouses. 

## 2. Materials and Methods

### 2.1. Materials

Methyl methacrylate (99%), N, N-dimethylformamide (99.9%), graphene oxide (4 mg/mL dispersion in water), copper(I) bromide (98%), copper (II) bromide (99%), 1,1,4,7,7-pentamethyldiethylenetriamine (98%), tetrahydrofuran (99.8%), zinc acetate dihydrate (99%), and ammonium hydroxide solution (25–28%) were purchased from FineLab Scientific Limited (Hong Kong). Triethylamine (≥99%), α-bromoisobutyryl bromide (98%), chloroform (containing 100–200 ppm amylenes as a stabilizer, ≥99.5%), aluminium chloride hexahydrate (ReagentPlus, 99%), and manganese (II) chloride tetrahydrate (ReagentPlus, ≥99%) were bought from Tin Hang Technology Limited (sole distributor of Sigma-Aldrich Inc. in Hong Kong). Butan-2-ol (99%), L-Ascorbic acid (98%), and acetic acid (AR 99.5%) were obtained from TiV Scientific Limited. 

### 2.2. Experimental

**Figure 1:**
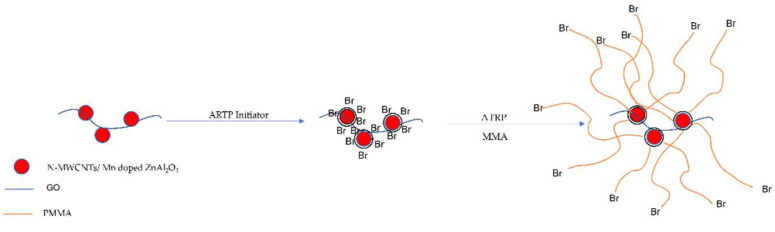
The scheme of surface-initiated atom transfer radical polymerization.

Figure 1 shows the scheme of this experiment. Briefly, ZnAl_2_O_4_:Mn is located on the surface of graphene oxide (GO), and then the -Br atom is added to the surface of ZnAl_2_O_4_:Mn/GO, which can initiate the polymerization, leading to a homogenous photoluminescent polymer composite.

#### 2.2.1. Fabrication of Photoluminescent ZnAl_2_O_4_:Mn/GO

A total of 10 g of Zn(COOCH_3_)_2_·2H_2_O and 22 g of AlCl_3_·6H_2_O (with the molar ratio of 1:2) were dispersed in 30 mL of deionized water by magnetic stirring. In the meantime, 0.54 g of MnCl_2_·4H_2_O was dissolved in 10 mL of DI water. Then, these two solutions were mixed by stirring for 2 h to make a homogeneous solution. Then, 30 mL of ammonium hydroxide was added to the mixed solution to change the pH value and form a white precipitate. The precipitate was washed with ethyl alcohol and DI water by centrifuging until the solution became neutral. The collected precipitate was preheated to 90 °C for 24 h before being sintered in air at different temperatures (1100 °C, 1200 °C, 1300 °C, 1400 °C, 1500 °C). 

A total of 2 mL of GO solution (8 mg) and 2 g of ZnAl_2_O_4_:Mn were dispersed in a mixed solution of 20 mL of CH_3_COOH and 7.5 mL of butanol. The mixture was stirred at 60 °C for 6 h. Then the solution was transferred into a 50 mL Teflon-sealed autoclave and then heated to 180 °C for 24 h. After the product was collected by suction filtration using a 0.2 μm filter membrane and washed with ethanol and DI water, the product was dried in a vacuum oven at 80 °C. 

#### 2.2.2. Synthesis of Photoluminescent Initiator ZnAl_2_O_4_:Mn/GO-Br

A total of 250 mg of ZnAl_2_O_4_:Mn/GO was dispersed in 15 mL of N, N-dimethylformamide (DMF) using an ultrasonic mixer. The mixture was transferred into a 50-mL two-neck spherical reaction bottle, which was pre-purged with nitrogen. Next, the whole reactor was purged with a vacuum and nitrogen for 30 min, and the purging cycle was repeated three times before beginning the reaction. Then, the reactor was put in an ice-water bath. After that, 4 mL of tetrahydrofuran (TEA) and 5 mL of α-bromoisobutyryl bromide were dropped into the reactor using an injection syringe. The reaction was kept at 0 °C for 3 h, and then the reaction was kept at room temperature for 48 h. After that, the product was collected by a vacuum filter through a 0.22 μm millipore polycarbonate membrane and washed with chloroform and acetone. Then, the product was dried under a vacuum at 70 °C overnight. 

#### 2.2.3. Polymerization of PMMA Composites and Pristine PMMA

Before polymerization, CuBr requires pretreatment. CuBr was dissolved in enough CH_3_COOH and stirred at room temperature overnight for purification. Then, the purified CuBr was washed with methyl alcohol, followed by drying in a vacuum oven at 40 °C for 12 h. It was then kept in a vacuum desiccator for further usage. 

A total of 0.736 g of ZnAl_2_O_4_:Mn/GO-Br, 0.088 g of CuBr, 0.152 g of CuBr_2_, and 0.036 g of L-ascorbic acid were dispersed in a mixed solution containing 10 mL of DMF and 4 mL of CH_3_COOH, and then 20 mL of MMA was added into the system. The whole system was purged by a vacuum-nitrogen cycle three times before the reaction. Then, 10 μL of extra α-bromoisobutyryl bromide and 142.4 μL of PMDETA were dropped into the reaction bottom using an injection syringe. The molar ratio of [MMA]:[-Br]:[CH_3_COH]:[PMDETA] = 574:1:324:2. After adding PMDETA, the whole system was heated to 90 °C and kept in an oil bath for 48 h. The molar ratio of [-Br] includes the -Br on the surface of phosphors/GO. 

Another sample with double the amount of phosphor was fabricated using the same method. A total of 1.472 g of ZnAl_2_O_4_:Mn/GO-Br r, 0.088 g of CuBr, 0.152 g of CuBr_2_, and 0.036 g of L-ascorbic acid were dispersed in a mixed solution containing 10 mL of DMF and 4 mL of CH_3_COOH, and then 20 mL of MMA was added into the system. The whole system was purged by a vacuum-nitrogen cycle three times before the reaction. Then, 10 μL of extra α-bromoisobutyryl bromide and 142.4 μL of PMDETA were dropped into the reaction bottom using an injection syringe. The molar ratio of [MMA]:[-Br]:[CH_3_COH]:[PMDETA]= 574:2:324:2. After adding PMDETA, the whole system was heated to 90 °C and kept in an oil bath for 48 h. 

The polymerization of pristine PMMA was similar to the process of polymer composites. A total of 0.088 g of CuBr, 0.152 g of CuBr_2_, and 0.036 g of L-Ascorbic acid were dispersed in a mixed solution containing 10 mL of DMF and 4 mL of CH_3_COOH, and then 20 mL of MMA was added into the system. The whole system was purged by a vacuum-nitrogen cycle three times before the reaction. Then, 40 μL of extra α-bromoisobutyryl bromide and 142.4 μL of PMDETA were dropped into the reaction bottom using an injection syringe. After adding PMDETA, the whole system was heated to 90 °C and kept in an oil bath for 48 h.

All the products were collected using a vacuum filter and purified through a dissolve filter process three times to remove extra ions. The purified composites were then dried at 80 °C for 48 h. The polymer sheet was produced by hot pressing at 145 °C. Then, the products were pressed into round pieces with a diameter of 50 mm and thickness of 1 mm at 150 °C with 30 KN pressure for further testing. 

### 2.3. Characterization

The structure and morphologies of products obtained from each step were tested using a scanning electron microscope (SEM, TESCAN VEGA3) and transmission electron microscope (TEM, Talos F200S). The photoluminescent properties were tested by a Hitachi F-7100-3D using a Xe lamp as the energy source. The Fourier-transform infrared spectroscopy (FTIR) spectrometers were recorded by Perkin Elmer with a wavenumber ranging from 500–4000 cm^−1^. The crystallization structures were analyzed through X-ray diffraction (XRD) (Rigaku Smartlab, Tokyo, Japan) scanning from 15° to 85°. The molecular weight and distribution of the polymer chain were detected by a Shimadzu Rid-20A (Kyoto, Japan), and the polymer was dissolved in tetrahydrofuran (THF) to make a solution of 20 mg/mL. Thermogravimetry-different thermal gravity (TG-DTG) was used to characterize the thermal properties of the polymer composites. Vickers hardness indentation was recorded by an Alicona Infinite Focus. 

## 3. Results and Discussion

### 3.1. Structure and Morphology

**Figure 2:**
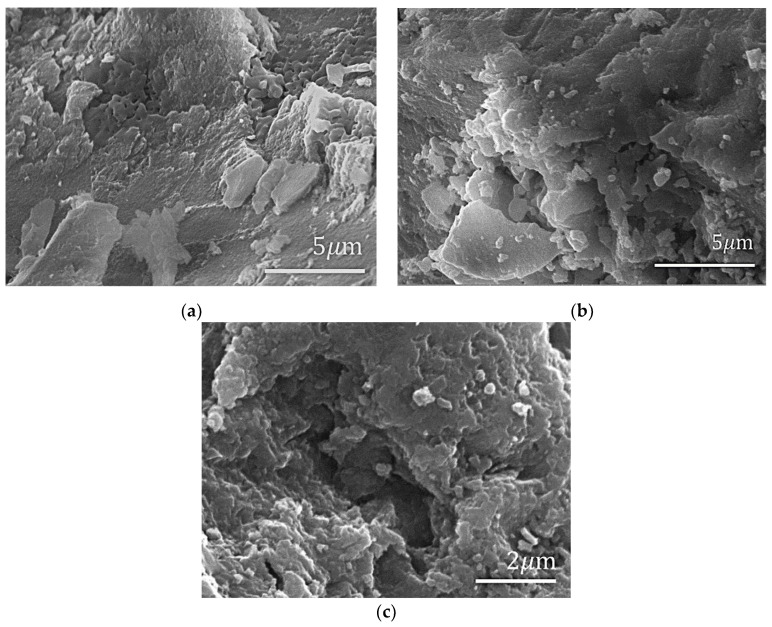
SEM micrographs of (**a**) ZnAl_2_O_4_: Mn annealing at 1200 °C; (**b**) ZnAl_2_O_4_: Mn annealing at 1300 °C; (**c**) ZnAl_2_O_4_: Mn annealing at 1400 °C.

Structure and morphology are crucial for the research of composites, and SEM micrographs were recorded and compared to analyze the morphological changes during each experimental step. Figure 2 shows the morphological changes of ZnAl_2_O_4_: Mn during the annealing process at different temperatures from 1000 °C to 1500 °C. Obviously, with the increase in annealing temperature, the particle size of ZnAl_2_O_4_:Mn becomes larger, and the layered structure of phosphors becomes clearer. The tendency is consistent with the result in the literature [30]. 

**Figure 3:**
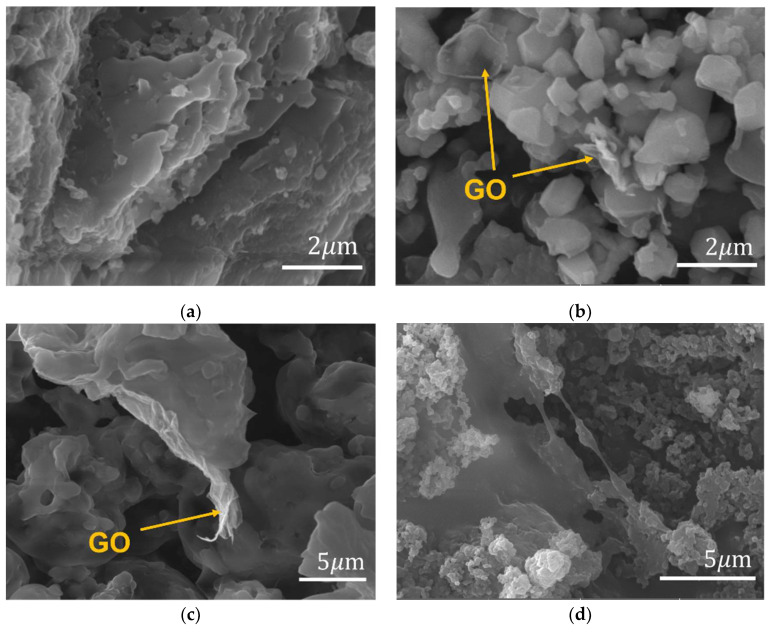
SEM micrographs of (**a**) ZnAl_2_O_4_: Mn annealed at 1400 °C; (**b**) ZnAl_2_O_4_: Mn/GO; (**c**) ZnAl_2_O_4_: Mn/GO-Br; (**d**) ZnAl_2_O_4_: Mn/GO-PMMA at 10kx magnification.

In this experiment, the colour change of each step is quite noticeable. Firstly, Mn-doped ZnAl_2_O_4_ was dark pink because of the oxidation of Mn during the annealing process, and then the outcome turned grey after the hydrothermal reaction because of the existence of GO. When -Br decorated ZnAl_2_O_4_: Mn/GO was collected, the product’s colour became a little bit light brown, which represents the colour of -Br. Figure 3 shows the morphologies of collected products after each experimental step. From Figure 3a,b, it can be seen that after being decorated by GO, the structure of photoluminescent ZnAl_2_O_4_: Mn changed to smaller layered pieces. The morphological changes are due to the interaction between ZnAl_2_O_4_: Mn and GO. Figure 3b,c show some transparent wrinkled structures on the surface of ZnAl_2_O_4_: Mn pieces, which may come from GO. Figure 3d shows the surface morphologies of polymer composite ZnAl_2_O_4_: Mn/GO/PMMA. In order to confirm the successful decoration of GO on the surface of ZnAl_2_O_4_: Mn, the structural and elemental information needs to be further studied by XRD and EDS. 

**Figure 4:**
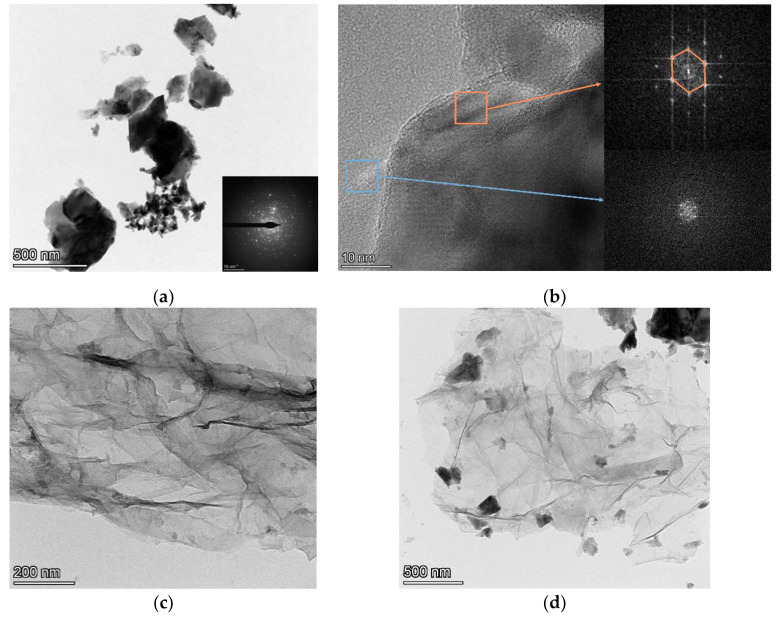
TEM micrographs and SEAD of (**a**) ZnAl_2_O_4_: Mn annealed at 1400 °C; (**b**) ZnAl_2_O_4_: Mn/GO; (**c**) GO; (**d**) ZnAl_2_O_4_:Mn/GO.

TEM micrographs are shown in Figure 4. Figure 4a shows the micrographs of ZnAl_2_O_4_ annealed at 1400 °C. The high-resolution TEM and SEAD results of Figure 4b show the hexagonal crystal structure of ZnAl_2_O_4__,_ which corresponds to the XRD results. Figure 4c shows the wrinkled graphene oxide thin layer, and combined with Figure 4d, it is clear that the phosphor Mn-doped ZnAl_2_O_4_ is successfully located on the surface of GO. 

**Figure 5:**
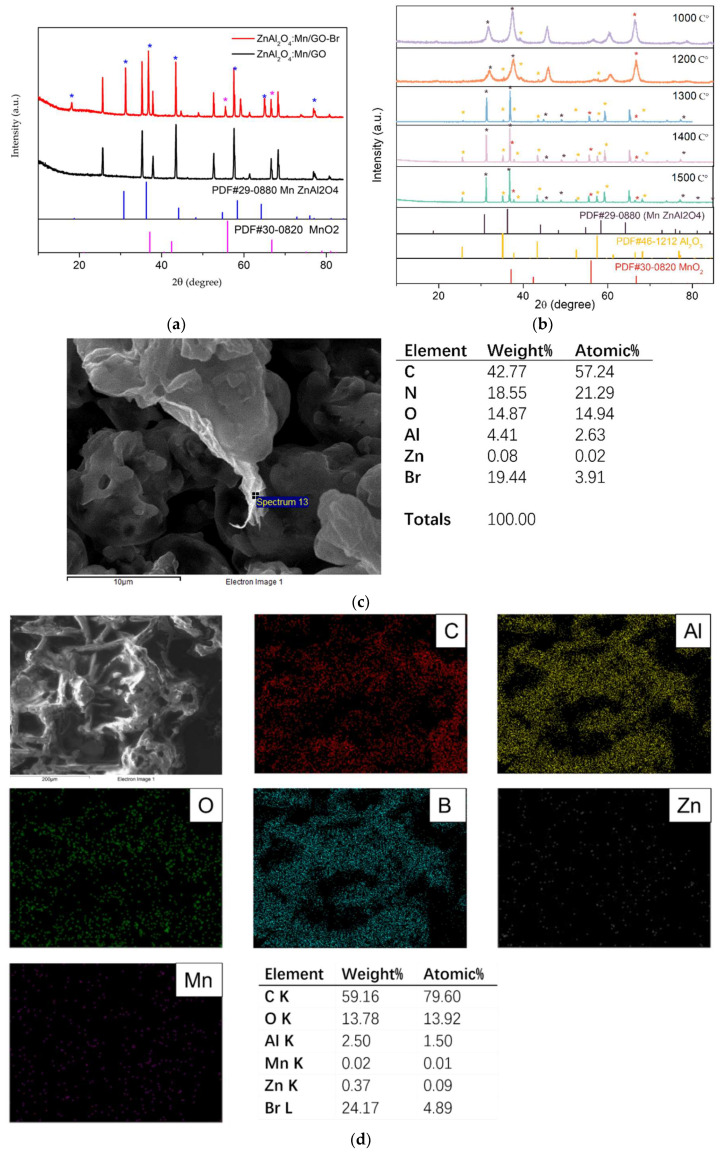
XRD of (**a**) ZnAl_2_O_4_: Mn/GO-Br and ZnAl_2_O_4_: Mn/GO; (**b**) ZnAl_2_O_4_: Mn annealed in air at different temperatures (1000 °C, 1200 °C, 1300 °C, 1400 °C and 1500 °C); (**c**) EDS of wrinkled GO; (**d**) EDS mapping of ZnAl_2_O_4_:Mn/GO-Br.

Figure 5a shows XRD patterns of phosphors annealed at different temperatures from 1000 °C to 1500 °C. The XRD spectra compare PDF#29-0880, PDF #46-1212, and PDF#30-8020 with the pdf cards of Mn ZnAl_2_O_4_, Al_2_O_3_, and MnO_2_. It shows that at 1000 °C, there are mainly peaks from Mn ZnAl_2_O_4_ at 32°, 37°, 46°, 55°, 60°, 75°, and 79°. Furthermore, when the temperature rises to 1200 °C, peaks representing Al_2_O_3_ at 35°, 39°, 43°, and 57° begin to be observed. Moreover, at 1300 °C, the peak intensity of Al_2_O_3_ becomes more vital. This proves that both ZnAl_2_O_4_ and Al_2_O_3_ exist in the final products when temperatures are higher than 1200 °C. Moreover, with the increase in temperature, the peak quality of Al_2_O_3_ is higher. 

Then, the XRD patterns of phosphors decorated by GO and Br are shown in Figure 5b below. XRD spectra of GO and reduced GO are referenced from L. Stobinski’s work [31], in which the main peak of graphene oxide is around 10°(2θ), while the prominent peak of reduced graphene oxide is around 26°. Therefore, the peak around 25° in Figure 5b should be from the reduced graphene oxide, which proves the reduction of graphene oxide during the transfer reaction of the active initiating part from α-bromoisobutyryl bromide to GO. The peak of rGO also proves the existence of GO. Compared with PDF#29-0880 and PDF#30-0820 shown in Figure 5a, for the pdf cards of Mn ZnAl_2_O_4_ and MnO_2_, most of the peaks are from them, which means that Mn was doped in the ZnAl_2_O_4_ structure, and the rest of Mn was oxidized to MnO_2_. Figure 5c records the EDS of ZnAl_2_O_4_:Mn/GO. The elemental dispersity of the wrinkled structure on the surface of ZnAl_2_O_4_:Mn shows a 57.24% C atom on the tested surface. Figure 5d shows the mapping EDS result of ZnAl_2_O_4_:Mn/GO-Br; the result shows the successful location of -Br on the surface of phosphors/GO. Considering the XRD result, it can be confirmed that the transparent structure is GO. Therefore, the SEM and XRD results prove the successful decoration of GO on the surface of phosphors. 

**Figure 6:**
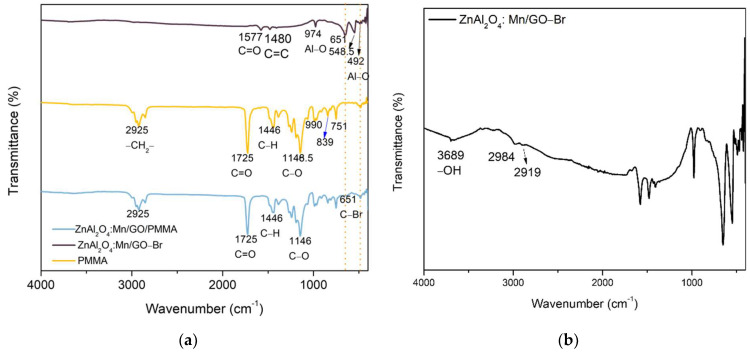
FTIR of (**a**) ZnAl_2_O_4_:Mn/GO-Br, PMMA and ZnAl_2_O_4_:Mn/GO/PMMA; (**b**) ZnAl_2_O_4_:Mn/GO-Br.

The initiation of ATRP begins after the formation of carbon radicals from the initiator, and then the carbon radical reacts with the −C=C− bond of the monomers. The oxygen functional groups on the GO surface will make the initiating reaction of carbon radicals easier because the electron-drawing functional groups are beneficial to the formation of the C-Br active group, which is the former of carbon radicals [32]. The ATRP initiator EBiB was reacted with GO first, and then the active group was transferred from EBiB to the surface of GO to begin the process of the above-mentioned initiating reaction and halide atom transfer process. In this reaction, CuBr was used as the catalyst, CuBr_2_ was used to keep the concentration balance of Cu(I) and Cu(II) in the reaction system, and α-bromoisobutyryl bromide was the initiator, which was covalently connected to the surface of GO, while PMDETA was a ligand. The covalent connection can be observed from the FTIR spectra in Figure 6 below.

FTIR for ZnAl_2_O_4_:Mn/GO-Br, ZnAl_2_O_4_: Mn/GO-Br/PMMA, and PMMA is shown in Figure 6. The FTIR results prove that the active group of ATRP -Br is located on the surface of GO. The FTIR pattern of ZnAl_2_O_4_: Mn/GO-Br displays a peak at 651 cm^−1^, representing C-Br, which reveals the existence of a connection between GO and the active group, -Br. The peaks at 1577 cm^−1^ and 1480 cm^−1^ correspond to the vibration of O-C=O and C-H of GO. Compared with PMMA polymerized through ATRP, the weak peak at 651 cm^−1^ reveals the formation of the active group, C-Br, during the polymerization from ZnAl_2_O_4_: Mn/GO-Br to the polymer chain. Figure 6b is the FTIR of ZnAl_2_O_4_: Mn/GO-Br, which is clearer to reveal the structure of the product; the peak at 3689 cm^−1^ refers to the -OH functional groups on GO. Peaks at 1577 cm^−1^ and 1480 cm^−1^ refer to C=O cm^−1^ and C=C cm^−1^ of GO. In conclusion, the FTIR result consists of the SI-ATRP mechanism.

### 3.2. Thermal Stability

**Figure 7:**
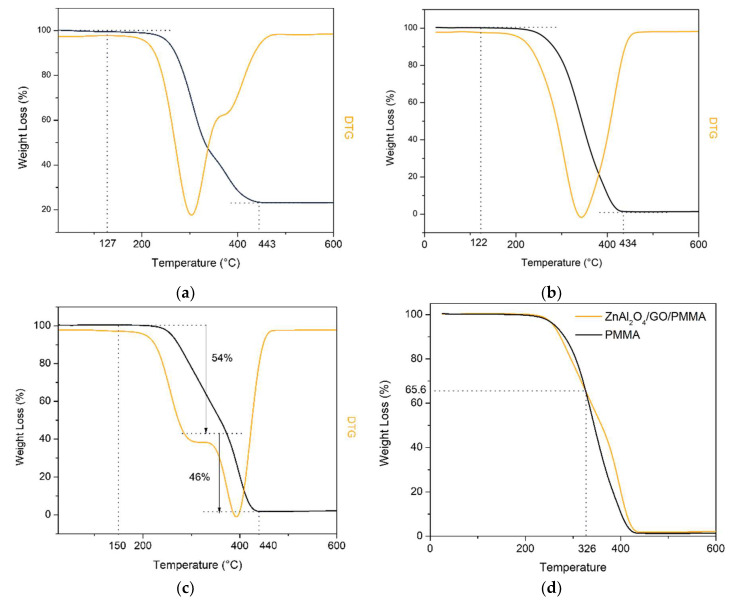
TG of (**a**) ZnAl_2_O_4_: Mn/GO-Br, (**b**) PMMA; (**c**) ZnAl_2_O_4_: Mn/GO/PMMA; (**d**) ZnAl_2_O_4_: Mn/GO/PMMA and PMMA.

The thermal stabilities of ZnAl_2_O_4_:Mn/GO-Br, PMMA, and ZnAl_2_O_4_: Mn/GO/PMMA were tested by TG-DTG. From the TG results in Figure 7, PMMA began to lose its weight at 122 °C, and it was decomposed entirely at 434 °C. In comparison, the polymer composite began to lose weight at 150 °C, which is 28 °C improved compared with PMMA, until 330 °C, and the composite lost 54% of its weight. The polymer composite ZnAl_2_O_4_: Mn/GO/PMMA was removed at 443 °C, which is 9 °C higher than for PMMA. Moreover, for the initiator, ZnAl_2_O_4_: Mn/GO-Br, the weight loss began at 127 °C, and it was decomposed completely at 443 °C. After the completion, it could be found that the thermal stability of polymer composite was a little bit higher than for pure PMMA but not evidently due to the limited addition of GO. The additive does not sacrifice the thermal stability of PMMA.

### 3.3. Molecular Weight

**Figure 8:**
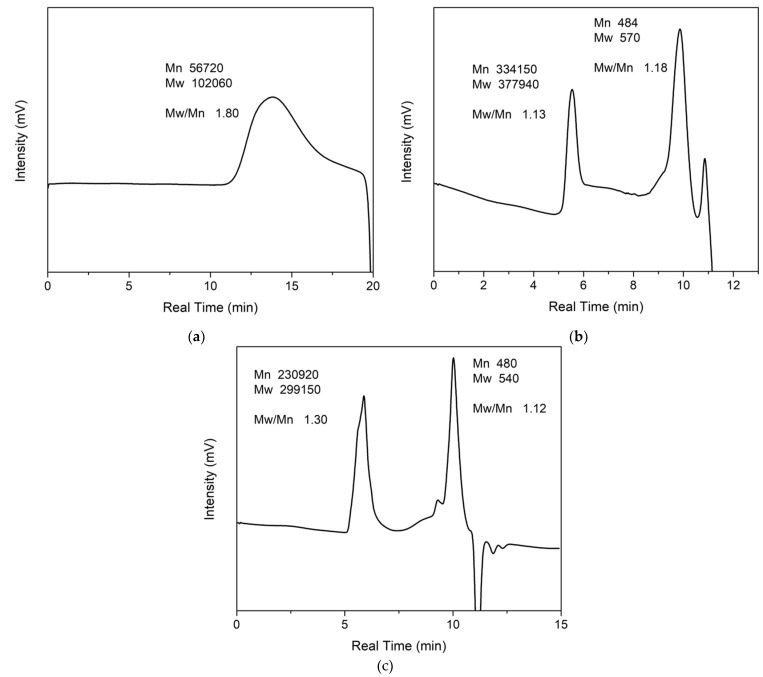
GPC of (**a**) PMMA; (**b**) double concentration ZnAl_2_O_4_:Mn/GO/PMMA; (**c**) ZnAl_2_O_4_: Mn/GO/PMMA.

GPC recorded the molecular weight of PMMA and ZnAl_2_O_4_: Mn/GO/PMMA with different concentrations of phosphor, and the tested polymer and polymer composites were dissolved in THF. Figure 8a shows the PMMA trace of GPC, and Figure 8b,c show the polymer traces of PMMA composites with different concentrations of phosphor. Figure 8b,c have two peaks, where the left peak should belong to polymer chains with a larger molecular weight, and the right peak should refer to the initiator with a small polymer molecular weight of around 500. 

The results are shown in Table 1 below. The number-average molecular weight (M_n_) of PMMA is 56,720, the mass average molar mass (M_w_) is 102,060, the molecular ion peak (M_P_) is 86,040, and the average molecular weight (M_z_) is 178,120. Therefore, the dispersity can be calculated by M_w_/M_n_ = 1.80. The dispersity of the ATRP PMMA is 1.80. The dispersity may be caused by the long polymerizing time of 48 h. In order to obtain a narrow dispersity, less reaction time is needed to control the active group number. Another possibility is the proportion between the initiator and monomers—the larger the proportion, the wider the molecular weight dispersity due to a large number of active groups. The GPC results of polymer composites show that the M_w_ is 377,940, which is 3.70 times that of pure PMMA with a narrow dispersity of 1.13. The difference in the initiator quantity causes this result. During the fabrication of PMMA, the initiator is only α-bromoisobutyryl bromide. The proportion of initiator and monomer is definite. However, when the initiator is transferred to the surface of GO, the proportion is not 100%, which leads to fewer initiators during the polymerization of polymer composites. Therefore, the proportion between initiator and monomer is less than for pure PMMA. Moreover, the dispersity is narrower than for pure PMMA due to the same reason. The narrow dispersity of polymer composites caused the homogeneous properties of polymer composites.

### 3.4. Photoluminescent Properties

**Figure 9:**
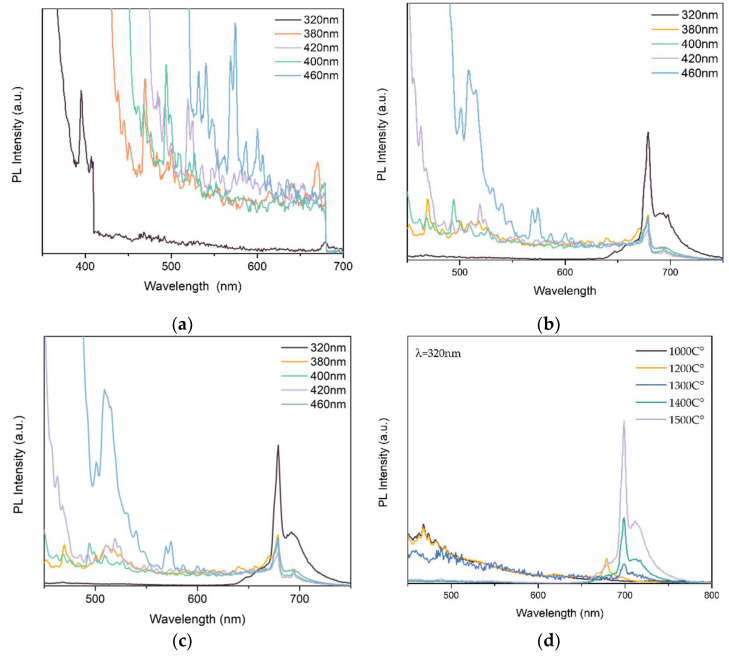
PL spectra of (**a**) ZnAl_2_O_4_: Mn annealed at 1300 °C; (**b**) ZnAl_2_O_4_: Mn annealed at 1400 °C; (**c**) ZnAl_2_O_4_: Mn annealed at 1500 °C; (**d**) ZnAl_2_O_4_: Mn annealed at different temperatures excited by 320 nm.

Figure 9 shows the PL spectra of ZnAl_2_O_4_:Mn annealed in air at different temperatures from 1300 °C to 1500 °C. Earlier research reported that Mn^2+^ behaves as an activator in the tetrahedral site of AB_2_O_4_ structural compounds [30]. The same results are shown in Figure 9 below. With the increase in exciting resource wavelength, a stronger green light was excited, with the exciting peaks at around 510 nm showing the process of electron-hole pair recombination from the ^4^T_1_ to ^6^A_1_ state of Mn^2+^ ion located in the tetrahedral site of Zn^2+^ within the spinel structure of ZnAl_2_O_4_ with a low crystal field strength.

As shown in Figure 9d, when the sintering temperature ranged from 1100 °C to 1500 °C, the excited green light became weak, and a stronger red light was excited. At a higher temperature, Mn^2+^ was doped into a rich-Al_2_O_3_ structure in ZnAl_2_O_4_ compounds, which was proved by XRD. With the quality increase in the Al_2_O_3_ structure, Mn^2+^ could activate stronger red light. Furthermore, with the increase in annealing temperature, oxidation of Mn^2+^ to Mn^4+^ occurred, as mentioned in XRD. Mn^4+^ can replace Al^3+^ in ZnAl_2_O_4_ compounds at high temperatures due to its similar radius and activate the red luminescence as the emission centre [10].

**Figure 10:**
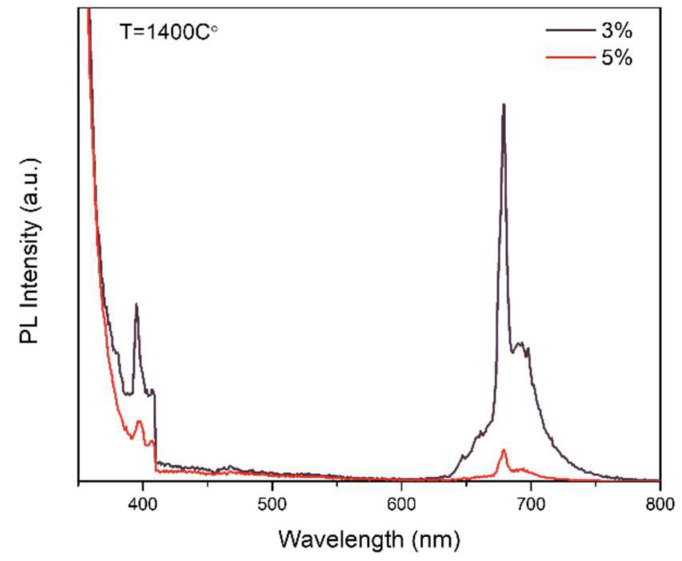
ZnAl_2_O_4_ was doped with different concentrations of Mn annealed at 1400 °C.

When the concentration of Mn rises from 3% to 5%, as Figure 10 shows, the emission is reduced, which is caused by the quenching of photoluminescence. In the structure, the Mn ion is located in the Zn ion tetrahedral coordination position in the ZnAl_2_O_4_ spinel structure. However, in host crystals, over-adding Mn ions will lead to inhomogeneous distribution from the emission centre [11].

To analyze the red and green light-emittance of the photoluminescent agents, 510 nm and 679 nm were selected as the monitoring wavelength to test the PLE spectra. The PLE of ZnAl_2_O_4_: Mn annealed at 1400 °C was recorded by monitoring the 510 nm emission band. The result shows three prominent peaks at 387 nm, 427 nm, and 455 nm, which are consistent with the electron transition of Mn^2+^ in the host lattice from ^6^A_1_ to ^4^T_2_(D), ^4^A_1_(G)/^4^E(G) and _4_T_2_(G), respectively [30]. Additionally, from PLE monitored by 679 nm, the 320 peak refers to the electron transition of Mn^2+^ from ^6^A_1_ to ^4^E(D), and the peak at 467 nm refers to the electron transition of Mn^4+^.

**Figure 11:**
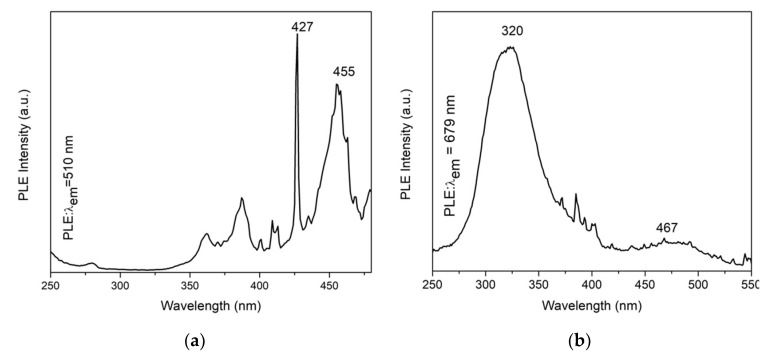

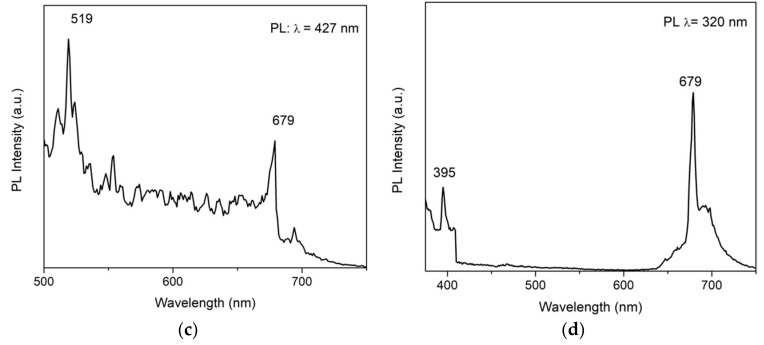
(**a**) PLE spectrum of ZnAl_2_O_4_:Mn annealed at 1400 °C at 510 nm; (**b**) PLE spectrum of ZnAl_2_O_4_:Mn annealed at 1400 °C at 679 nm; (**c**) PL spectrum of ZnAl_2_O_4_:3Mn annealed at 1400 °C and excited by 427 nm; (**d**) PL spectrum of ZnAl_2_O_4_: Mn annealed at 1400 °C and excited by 320 nm.

Figure 11a shows the most substantial peak at 427 nm. When 427 nm was selected as an excitation source, strong peaks at around 519 nm were excited, showing the electron-hole pair recombination process from the ^4^T_1_ to ^6^A_1_ state of the Mn^2+^ ion. When 320 nm was selected as the exciting source, a peak at 679 nm was recorded, referring to the recombination of Mn^4+^.The results are consistent with literature [10,11,30].

**Figure 12:**
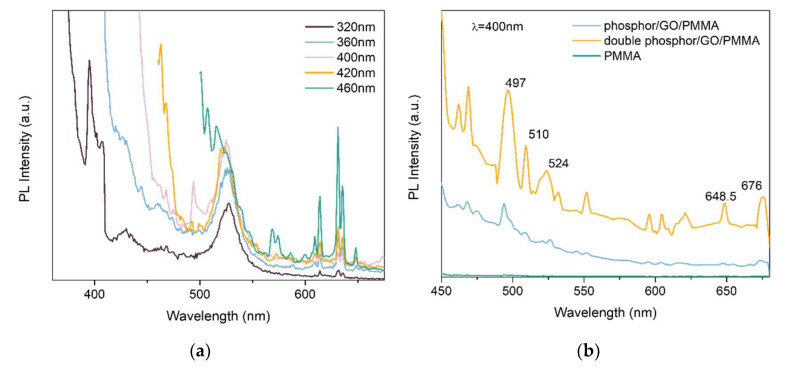

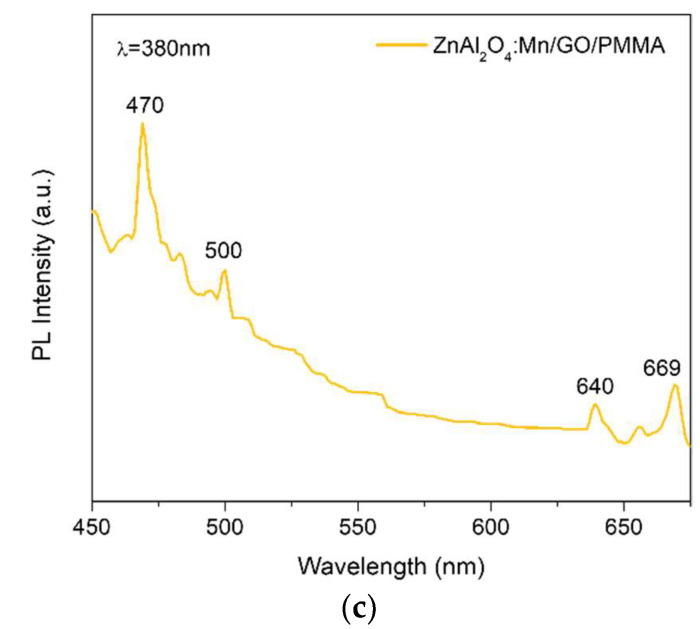
(**a**) PL spectra of ZnAl_2_O_4_: Mn/GO-Br; (**b**) PL spectra of PMMA and ZnAl_2_O_4_: Mn/GO/PMMA excited by 420 nm; (**c**) PL spectrum of ZnAl_2_O_4_:Mn/GO/PMMA excited by 380 nm.

Figure 12 shows the PL spectra of the phosphor, ZnAl_2_O_4_:Mn/GO, which was modified by initiating the active group and the polymer composite, ZnAl_2_O_4_:Mn/GO/PMMA, with different concentrations of phosphors which were fabricated through SI-ATRP. Figure 12a shows the PL spectra of the modified phosphor excited by different wavelengths. From the picture, it can be observed that due to the reduction of GO during the initiating active group transfer reaction, the photoluminescent quenching from graphene oxide was avoided. If there are too many oxidation groups on the surface of GO, the excited electrons will be transferred to the surface GO instead of going through the luminescent process. Figure 12b shows the PL spectra of polymer composites with different concentrations of phosphors and pure PMMA. There is an apparent green light and red light excited by the source with a wavelength of 420 nm from ZnAl_2_O_4_:Mn/GO compared with PMMA. Additionally, with the increase in phosphor concentration, the excited green light and red light become more obvious. Figure 12c shows the PL spectrum of ZnAl_2_O_4_: Mn/GO/PMMA excited by 380 nm, and in this spectrum, both green light and red light were excited. The results show that the photoluminescent property of polymer composites is related to the concentration of phosphors. 

### 3.5. Hardness Testing

**Figure 13:**
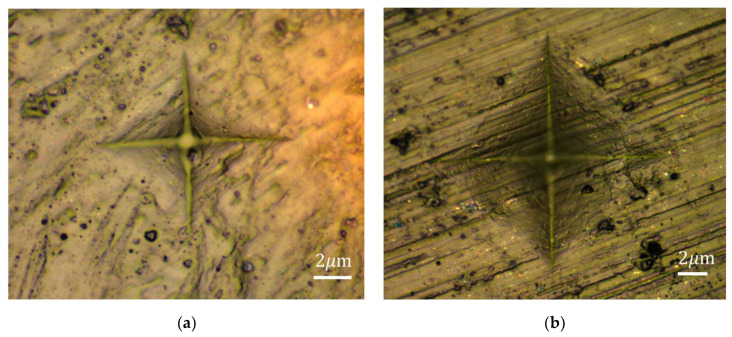
Vickers hardness indentation of (**a**) PMMA; (**b**) ZnAl_2_O_4_: Mn/GO/PMMA.

The hardness of the pure polymerized PMMA and the photoluminescent polymer composites were tested through the Vickers hardness method. Figure 13 shows two examples of the indentation of PMMA and PMMA composites after the Vickers hardness experiment. Each species was tested for 20 points. The average Vickers hardness of PMMA is 51.42 HV, while the average Vickers hardness of polymer composites is 49.62 HV. From the figure, it could be found that with the addition of ZnAl_2_O_4_:Mn/GO, the hardness reduces slightly. The addition of ZnAl_2_O_4_:Mn/GO does not reduce the hardness much compared with pure PMMA, so the addition of phosphors does not sacrifice the hardness of the material.

## 4. Conclusions

In this research, homogeneous ZnAl_2_O_4_:3% Mn/GO/PMMA polymer composites with narrow dispersity (1.13) were successfully synthesized through SI-ATRP. The polymer composites have green and red dual light-emitting properties when excited by 400 nm, which can be used for LED lampshades. Compared with widely used physical methods, this research fabricated controllable molecular weight and homogeneous polymer composites with narrow dispersity. Mn-doped ZnAl_2_O_4_ could be excited as green and red dual light with one wavelength and could be synthesized under relatively simple conditions. In addition, ZnAl_2_O_4_:Mn is environmentally friendly and economical, which is a promising replacement for photoluminescent material of rare-earth metal ion-doped materials. GO is used as the bridge between phosphors and polymer chains because the polymerization is initiated by the Br on the GO surface. The existence of GO could also improve the mechanical properties of polymer composites. PMMA is a widely used polymer material with good optical properties. After polymerization with phosphor, the narrow-dispersed polymer composites can be machined directly on the surface without coating. This will give the polymer composite materials commercial potential in plant factories and greenhouses. 

## Figures and Tables

**Table 1:** GPC results of PMMA and ZnAl_2_O_4_:3%Mn/GO/PMMA composites.

Sample	M_n_	M_w_	M_z_	M_z+1_	Dispersity	M_z_/M_w_
PMMA	56,720	102,060	178,120	274,040	1.80	1.74
ZnAl_2_O_4_: Mn/GO/PMMA	334,150	377,940	420,400	461,030	1.13	1.11
Higher concentration ZnAl_2_O_4_: Mn/GO/PMMA	230,920	299,150	357,550	411,200	1.30	1.20
